# Use of gastroscopy in the management of pediatric toxic ingestions: insights from a decade of experience – a retrospective study

**DOI:** 10.3389/ftox.2026.1678559

**Published:** 2026-02-12

**Authors:** Marco Marano, Lavinia Di Meglio, Mara Pisani, Bianca Maria Goffredo, Carla Olita, Simona Faraci, Francesca Rea, Salvatore Perdichizzi, Giorgio Zampini, Piero David, Filippo Torroni

**Affiliations:** 1 Pediatric Poison Control Center, Bambino Gesù Children’s Hospital, IRCCS, Rome, Italy; 2 Pediatric Intensive Care Unit, Bambino Gesù Children’s Hospital, IRCCS, Rome, Italy; 3 School of Pediatrics, University of Rome Tor Vergata, Rome, Italy; 4 Emergency, Acceptance and General Pediatrics Area, Bambino Gesù, Children’s Hospital, IRCCS, Rome, Italy; 5 Division of Metabolic Biochemistry, Bambino Gesù Children’s Hospital, IRCCS, Rome, Italy; 6 Digestive Endoscopy and Surgery Unit, Bambino Gesù Children’s Hospital, IRCCS, Rome, Italy

**Keywords:** endoscopic decontamination, esophagogastroduodenoscopy, gastrointestinal decontamination, pediatric toxicology, pharmacobezoar, sustained-release formulations, toxic ingestion

## Abstract

**Objective:**

Pediatric poisonings account for a significant proportion of poison center consultations. Gastrointestinal decontamination (GD) is a crucial intervention in cases of acute poisoning, yet its effectiveness remains debated. This study aims to evaluate the role of esophagogastroduodenoscopy (EGD) in pediatric GD by retrospectively analyzing cases of suspected or confirmed toxic ingestions in which EGD was employed as a decontamination technique.

**Methods:**

A retrospective review was conducted on medical records from our hospital between 1 January 2015, and 31 March 2025. Pediatric patients (<18 years) who underwent EGD for GD following suspected or confirmed ingestion of toxic drugs were included.

**Results:**

A total of 19 cases met the inclusion criteria. In all cases, a potentially toxic drug dose was ingested. EGD was primarily indicated in the presence of sustained-release (SR) formulations, delayed gastric emptying, or severe clinical presentation. In 15 cases, xenobiotic residues (pharmacobezoars, intact tablets, or gastric contents containing drug material) were successfully removed via EGD. No complications were reported, and all patients had favorable outcomes.

**Conclusion:**

EGD appears to be an effective GD technique in selected pediatric poisoning cases, particularly those involving bezoar-forming drugs, SR formulations, and substances that impair gastrointestinal motility. It may also be useful in cases of massive ingestion of potentially harmful substances. Further research is needed to establish standardized guidelines for its use in pediatric toxicology.

## Introduction

### Background

Pediatric exposures account for nearly 56% of human exposure calls to U.S. poison centers, according to the latest 2023 data. Among these, 39.8% involve children under the age of five. While pediatric poisonings are primarily unintentional—often resulting from general accidental exposures, therapeutic errors, or unintentional misuse—intentional exposures become the leading cause among adolescents (ages 13–19) and adults (Gummin et al., 2024).

Gastrointestinal decontamination (GD) plays a critical role in managing poisoned patients. Recent U.S. data from 2023 indicate that 78.23% of human exposures occur via the gastrointestinal (GI) route, underscoring the importance of GD in acute poisoning management (Gummin et al., 2024; Albertson et al., 2011; Ornillo and Harbord, 2020).

The most commonly used GD techniques include gastric lavage (GL), activated charcoal (AC), cathartics, whole bowel irrigation (WBI), induced emesis, esophagogastroduodenoscopy (EGD) in selected cases, and combined approaches (Table 1) (Gosselin et al., 2025; Smith, 2010). However, the utility of these techniques remains controversial in the literature, particularly following the most recent position statements. Their effectiveness is often supported only by small studies and lacks robust evidence-based validation (American Academy of Clinical Toxicology and European Association of Poisons Centres and Clinical Toxicologists, 2005; Benson et al., 2024; Höjer et al., 2013; Thanacoody et al., 2015; Park et al., 2018; Hoegberg et al., 2021; Sandilands, 2023). In clinical practice, there is still no clear consensus, and adherence to existing guidelines remains low. Moreover, the use of these techniques has declined over the past decade. According to the 2023 Annual Report, AC was used in only 0.42% of pediatric exposures, while ipecac syrup and GL were not used at all, and WBI was used in only 0.01% of cases (Gummin et al., 2024).

**TABLE 1:** GD techniques.

GD techniques	Procedure	Indication	Contraindication
GL	Position of a Levin probe (32–36 French in adults, 22–28 French in children) and the infusion and aspiration of large volumes of water (almost 2 L)	Within 1 hour from ingestion with trained medical operators	>1 h from ingestion for lack of benefit Craniofacial anomalies Unprotected airways Untrained medical operators. Caustic Previous esophagus surgery
AC	Charcoal is obtained by controlled pyrolysis of coconut shells, peat, lignite, wood, or petroleum, the activation comes form heating in steam, air, or carbon dioxide at 600 °C–900 °C. This process creates a surface area of 950 to 2,000 m2/g The dosage ranges from 0.5 g/kg to 1 g/kg however, a higher dose may be needed depending on the drug ingested. Multiple dose are needed in case of xenobiotics with enterohepatic circulation	Within 1 hour from ingestion of drugs absorbed by AC, however, for slow releaser AC can be used up to 4 h	Drugs not absorbed by AC. >1 h from ingestion or 4 h in case of slow releaser Unprotected airways Allergy to AC Caustic
WBI	Enteral administration of an osmotically balanced polyethylene glycol-electrolyte solution to enhance bowel emptying and to reduce the drug absorption	Potentially toxic ingestions of sustained-release or enteric-coated drug, drugs not adsorbed by AC (e.g., lithium, potassium, and iron), and for removal of illicit drugs in body “packers” or “stuffers	Severe abdominal distension, vomiting Unprotected airways for the risk of aspiration Allergy Perforation
Ipecac syrup	Oral ingestion of ipecac syrup which contains emetine and cephaeline that induce emesis by local gastric irritation and by the stimulation of the emesis chemo-trigger zone	No indicated according to the last AACT and EAPCCT position statement	​
EGD Not included in the AACT and EAPCCT position statement*	Use of an endoscope adapted to the patient’s characteristics	Bezoars SR	Unprotected airways Anatomical esopaghus anomalies Untrained medical operators

Conducted an international, multicenter, cross-sectional prospective study analyzing the use of GD in children under 18 years of age with toxic exposures. The study assessed the appropriateness of these interventions and found that GD procedures were deemed inappropriate in more than 50% of cases (Mintegi et al., 2017).

Over the past decade, several case reports have described the use of EGD as a GD technique in the management of severe or refractory poisonings, particularly those involving sustained-release (SR) formulations or drugs with a tendency to form bezoars (Gosselin et al., 2025). In this retrospective study, we analyzed all pediatric cases of suspected or confirmed toxic drug ingestion managed at our hospital—with the support of our dedicated pediatric poison control center—in which EGD was used as a decontamination technique.

## Methods

### Ethics

This study was conducted in accordance with the Helsinki Declaration (Fortaleza revision, 2013), Good Clinical Practice Standards (CPMP/ICH/135/95), and the current Decree-Law 196/2003 regarding personal data protection, as well as all applicable European regulations on this subject. This study was approved by the Ethics Committee of Bambino Gesù Pediatric Hospital code. n. 3648/2025 and date of approval 11/07/2025. Informed consents were collected in all patients.

### Study design, setting and selection of participants

We retrospectively analyzed all medical records from the past 10 years, covering the period from 1 January 2015, to 31 March 2025. We selected all pediatric patients with suspected or confirmed ingestion of a toxic drug dose who were treated at our hospital. The inclusion criteria were:

- Confirmed or suspected ingestion of a potentially toxic drug dose.
- Age <18 years.
- Undergoing EGD as a decontamination technique.

The exclusion criterion was:

- Age >18 years.

### Interventions and measurements

The indication for EGD at our center was based on multiple factors:

- Factors related to the drug: ingestion of medications at toxic doses with potential for significant harm, ingestion of sustained-release formulations, or drugs known to delay gastric emptying or reduce gastrointestinal motility due to anticholinergic activity.
- Factors related to the patient: the clinical condition of the patient and the anticipated severity of toxicity.
- Factors related to previous experience: previous experience at our institution and evidence reported in the literature.

For each patient, we collected data on the following variables: age, ingested drug (s) and dosage (if known), time between ingestion and arrival at the emergency unit (EU), other GI decontamintion techniques performed, Poisoning Severity Score (PSS), clinical and laboratory abnormalities, indication for EGD, time between ingestion and EGD, EGD findings, complications of the procedure, serum drug levels (if available), and clinical outcomes. The dosage assumed and the serum drug level were considered toxic using the values proposed by Micromedex. EGD was performed by expert pediatric endoscopists under general anaesthesia with advanced airway control. The procedure was conducted either in the operating room or the pediatric intensive care unit (PICU), using an appropriate endoscopic tool (Olympus GIF H 190–185 Tokyo, Japan). The procedures included gastric irrigation with saline solution 0.9%, accurate removal of gastric material using a specific device (retrieval devis Boston Scientific) and aspiration. Informed consent was obtained from all patients’ legal guardians before the procedure. Following gastroscopy, all patients underwent continuous 24-h monitoring.

### Outcome

All analyzed variables were collected and organized into a comprehensive table. For each patient, we assessed the ingested dose, the time interval between ingestion and endoscopy, available blood levels, endoscopic findings, final clinical outcome, and any adverse effects or complications related to the procedure.

### Data collection and analysis

Two authors (LDM, MM) reviewed all clinical records and extracted the data.

## Results

The results are presented in Table 2. We analyzed all the medical records of the last decade and 19 cases met the inclusion criteria.

**TABLE 2:** Clinical, laboratory, and EGD features of pediatric patients with toxic syndrome who underwent EGD as a GD techniques.

n	Age/Sex weight	Drugs (plasmatic level)	Toxic dose	Clinical condition and exams	PSS	Hours before EGD	EGD
01	15 years/F 55 kg	lithium sulfate SR 2490 mg (3.37 mmol/L)	>2.5 mmol/	drowsiness, confusion metabolic acidosis, >AST, ALT	**3**	17	negative
02	15 years/F 70 kg	-isoniazid 30gr (93.45 μg/mL) -rifampicin 2400 mg (unknown)	−40 mg/kg -unknown	vomit, coma, convulsions, metabolic acidosis, > AST, ALT and CPK	3	12	bezoar
03	16 years/M 51 kg	eltrombopag 4200 mg (188 mcg/mL)	Unknown	drowsiness and confusion, esophagus erosion	2	2.5	tablet residual
04	14 years/F 95 kg	quetiapine SR 1400 mg (unknown)	>10 mg/kg	drowsiness and confusion >QTc, > pancreatic enzymes	2	3	Gastric material with residual tablets
05	17 years/F 56 kg	-biperiden 160 mg (unknown) -lithium sulfate SR 1162 mg (1.37 mmol/L)	-unknown >2.5 mmol/L	drowsiness and confusion	2	2	tablet residual
06	17 years/F 50 kg	Aripiprazole 125 mg (unknown)	90 mg	>CPK	1	3	tablet residual
07	13 years/M 72 kg	lithium sulfate SR 4150 mg (2.7 mmol/L)	>2.5 mmol/L	Vomit, extreme agitation >AST, ALT, CPK (>10′000U/l), AKI	**3**	11	Gastric material with residual tablets
08	13 years/F 50 kg	acetylsalicylic acid 9750 mg (5.30 mg/dL)	150 mg/kg	none	0	4	bezoar
09	14 years/F 45 kg	-quetiapine SR uncertain mg (1784 ng/mL) -fluoxetine uncertain mg (235 ng/mL)	->10 mg/kg >1,200 mg	coma and hypotension metabolic acidosis	3	4	bezoar
10	16 years/M 75 kg	caffeine 14000 mg (53 mcg/mL)	−50–200 mg/kg	vomiting, metabolic acidosis, AKI and >CPK	3	5	negative
11	17 years/F 60 kg	-amitriptyline 112.5 mg (unknown) -chlordiazepoxide 112,545 mg (unknown)	->5 mg/kg ->2000 mg	drowsiness and confusion, ileus, bradycardia	3	6	tablet residual
12	17 years/F 108 kg	lithium sulfate SR 2905 mg (2.96 mmol/L)	>2.5 mmol/L	>AST, ALT	1	6	bezoar
13	11 years/F/104 kg	-repaglinide 15 g (unknown) -pioglitazone 450 mg (unknown) -metformin 25.5 gr (1 mg/L)	-4 mg -unknown >5gr	none	1	3	Gastric material with residual tablets
14	16 years/F/93 kg	-clozapina 1900 mg (unkown) -clotiapina 1,600 mg (unkown)	->62.5 mg -unknown	confusion >AST, ALT	2	3	Gastric material with residual tablets
15	13y F/43 kg	-sertraline 1,500 mg (unkown)	->2 gr	lethargy	2	2	bezoar
16	16y F/56 kg	Aripiprazole 1000 mg (791.10 ng/mL)	90 mg	Confusion mild metabolic acidosis	1	10	negative
17	15y F/70 kg	Carbamazepine 6500 mg (27.6 mcg/mL)	3.2 g 30 mcg/mL	Coma	3	28	negative
18	17y F/50 kg	-lurasidone 814 mg (unkown) -alprazolam SR 60 mg (unkown) -Lithium sulfate SR 830 mg (1.2 mmol/L) -biperiden hydrochloride 40 mg (unkown) -lorazepam 7.5 mg (unkown) -Melatonin 70 mg (unkown)	-unkown ->7.5 mg −2.5 mmol/L -unkown ->2gr -unkown	Sinus bradycardia drowsiness and confusion	2	>12 h	bezoar
19	17y M/ 64 kg	-Lithium sulfate SR 2407 mg (1.08 mmol/L) -Olanzapine SR 27,5 mg (29.89 ng/mL)	>2.5 mmol/L >100 ng/mL	>AST, ALT	1	6	tablet residual

In all cases, a potentially toxic dose of the drug was ingested. In cases 01, 02, 03, 05, 07, 09, 11, 13, 14, 15, 18, 19 the drug was part of the patient’s home therapy and was ingested in all cases with suicidal intent. GL was performed before EGD in all cases except 11, 13, 17, and 18. AC was administered before EGD in cases 02, 03,04, 05, and 10, and after EGD in cases 11, 15.

The indication for EGD was based on multiple factors, including:

- Certain or reported ingestions of medications at toxic doses that could potentially cause harm (01–19).
- The ingestion of sustained-release (SR) formulations (01, 04, 05, 07, 09, 12, 18, 19).
- Drugs known to delay gastric emptying or reduce GI motility due to their anticholinergic activity (06, 09, 11, 14, 15, 18, 19).
- The clinical condition of the patient and the potential toxicity (01–19).
- Previous experience from our hospital or present in literature (01, 04–07, 09, 11, 12, 14–16, 18, 19).

In cases where bezoars (defined as aggregates of inedible or undigested material), solid tablets, or food material were present, complete aspiration and removal of the xenobiotic were successfully performed.

**FIGURE 1:**
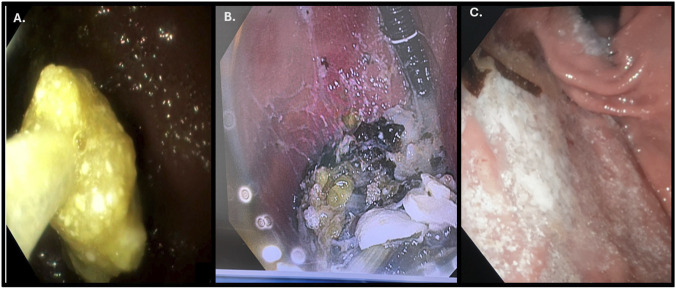
**(A)**. Bezoar; **(B)** Gastric material with residual tablets; **(C)** tablet residual.

EGD findings were negative in cases 01, 10, 16, and 17. Pharmacobezoars were observed in cases 02, 08, 09, 12, 15, and 18. Additionally, in cases 02 and 18, residuals in their reticular phase were also reported. Single tablets not forming bezoars were identified in cases 03, 05, 06, 11, 19; while gastric material with residual drug content was found in cases 04, 07, 13, and 14 (Figure 1).

Drug’s blood levels were obtained in all cases except for 04, 06, 11, 14, and 15. However, these results were only available after the procedure and were not useful in the initial management. Nevertheless, in all the performed cases, elevated drug levels were confirmed apart form case 05, 17, 18, and 19. The levels were monitored, and in all cases, a reduction and, eventually, an absent concentration were observed.

All patients were intubated for the procedure and extubated afterward, except for Patient 09, 10, and 17. Patient 9 was extubated after 48 h due to a comatose state and hemodynamic instability; a 72 h monitoring in PICU was needed. Patient 10 required continuous veno-venous hemodiafiltration (CVVHDF), was extubated after 24 h and needed a 48 monitoring in PICU. Patient 17 required intubation initially for a coma and after for respiratory failure due to a ventilator associated pneumonia and was extubated after 14 days. Additionally, the patient required CVVHDF and was assisted in PICU for 2 weeks.

Patients 01 were admitted to the PICU for the first 48 h and required additional treatments, including CVVHDF. Patients 02, 07, and 16 required 72 h of monitoring in the PICU. Patient 02 received pyridoxine and N-acetylcysteine due to elevated creatine phosphokinase (CPK) and transaminase levels.

Patients 03 and 12 were admitted to the Intensive Short-Term Observation Unit of the ED for 24 h for continued monitoring. Meanwhile, Patients 06, 11, 13, 14, 15, 18, and 19 were admitted to the PICU for 24 h.

Patients 05 and 08 were monitored in the pediatric department for the first 48 h.

Patient 4 was admitted directly in the Neuropsychiatry Unit (NP).

Following their initial admissions to either the pediatric unit or the PICU, all patients were subsequently transferred to the NP unit. The median length of stay in the NP unit was 6 days (range: 3–60 days).

The outcome was favorable for all patients, they were discharged home in good clinical condition. No adverse events were reported following the procedure.

## Discussion

The GI tract plays a fundamental role in drug absorption. The toxicokinetics and toxicodynamics of a xenobiotic—such as its anticholinergic properties, SR formulation, or propensity to form bezoars—along with individual patient factors (e.g., co-administration of other substances, anatomical variations, and time since last meal), determine the location and timing of absorption within the GI tract (Miyauchi et al., 2015).

In the stomach, xenobiotics progress through three phases: the tablet/food phase, the soluble/fluid phase, and the reticular/empty phase, each corresponding to a distinct stage of digestion (Miyauchi et al., 2015). Until absorption occurs, drugs technically remain outside the systemic circulation and thus cannot exert toxic effects (Nelson, 2000). The primary objective of gastrointestinal decontamination is to remove the xenobiotic as promptly as possible to prevent systemic absorption. As shown in Table 1, the effectiveness of techniques such as AC and GL is highly time-dependent. In practice, however, patients rarely present to the hospital within 1 hour of ingestion, and routine steps—including medical history collection, physical examination, and poison center consultation—inevitably delay intervention (Albertson et al., 2011; Ornillo and Harbord, 2020; Gosselin et al., 2025).

Although EGD is not currently included in standard guidelines for managing toxic ingestions, its use as a decontamination method was first proposed in 1986 (Gosselin et al., 2025). Initial supporting evidence stems from observational studies. For instance, Miyauchi et al. evaluated endoscopic findings after GL and found no clear correlation between the type of ingested drug and the time elapsed since ingestion (Miyauchi et al., 2015). Similarly, Livshits et al. identified whole tablets, fragments, granules, paste, or slurry in 9% of autopsies of fatal drug overdose cases—98% of which involved drugs known to delay gastric emptying, slow GI motility, or use SR formulations (Livshits et al., 2015).

In our retrospective study, EGD was used as a decontamination technique in 19 pediatric cases of suspected or confirmed toxic ingestion. In 15 of these cases, xenobiotic residues were identified and successfully removed, with no procedural complications reported.

The existing literature on decontaminative EGD is limited to small case series and individual reports. Table 3 summarizes 20 publications describing 32 cases of EGD-assisted decontamination. Similar to our findings, these studies found no consistent correlation between the timing of EGD and endoscopic findings. Common indications included radiographic suspicion or confirmation (via X-ray or CT) of residual tablets or bezoars in the stomach, severe clinical presentation, persistently elevated drug levels, and prior institutional or literature-based experience.

**TABLE 3:** Literature review of EGD use as a GD technique in toxic syndrome.

Ref.	Sex/Age	Drugs	PSS	Other GD	Hours before EGD	EGD/abdomen RX or TC	Outcome
Rauber-Lüthy et al. (2013)	40/F	Quetiapine 18 g Lorazepam	3	​	**>16h**	Bezoar	favorable
Rauber-Lüthy et al. (2013)	41/M/	Quetiapine 15gr Ethanol	3	​	13 h	Bezoar	favorable
Rauber-Lüthy et al. (2013)	28/M	Quetiapine 24gr Paliperidone SR Codeine Acetaminophen	2	​	2.5 h	Bezoar	favorable
Rauber-Lüthy et al. (2013)	22/M	Quetiapine 15 g Lornoxicam Acetylsalicylic acid	2	​	7.5 h	Bezoar	favorable
Rauber-Lüthy et al. (2013)	48/M	Quetiapine 15gr Velfalexina	3	​	>2 h	Bezoar	favorable
Rauber-Lüthy et al. (2013)	28/M	Quetiapine 6gr	3	​	2.5 h	Bezoar	favorable
Rauber-Lüthy et al. (2013)	26/F	Quetiapina Mirtazapine Lorazepam	3	​	**2.25h**	Bezoar	favorable
Rauber-Lüthy et al. (2013)	26/F	Quetiapina 3gr Lorazepam Venlafaxine SR Levomepromazine Metamizole	3	​	3–7 h	tables and food residuals	favorable
Rauber-Lüthy et al. (2013)	38/M	Quetiapine 11.8gr	3	​	2 h	Bezoar	favorable
Reuchsel and Gonnert (2022)	64/F	Quetiapine	3	​	>24 h	residual tablets/negative	favorable
Guillermo et al. (2014)	42/F	KCl 1,000 mEq SR clonazepam 50 mg	2	AC/GL	2–4 h	solid tablets/radiopaque image	favorable
Sato and Kamijo (2014)	49/M	Vegetamine A	3	AC	>72 h	Bezoar/ radiopaque image	favorable
Gavala et al. (2017)	43/M	Methadone	3	AC/WBI	>24 h	Bezoar/radiopaque image	favorable
Labarinas et al. (2018)	17/M	Diphenhydramine 20gr	3	AC/ WBI	>24	Bezoar partially removed/radiopaque image	favorable
McCarthy and Coleman (2023)	37/F	lamotrigine 2.6gr Clomipramine SR 4.2 gr	3	​	20 h	Bezoar/ radiopaque image	favorable
Allen et al. (2022)	14/M	Fluoxetine Clonidine	3	WBI	4 h	Solid tablets	favorable
Briggs and Deal (2014)	44/F	KCl SR 600 mEq Alprazolam 60 mg	3	WBI	6 h	Bezoar/radiopaque image	favorable
Höjer and Personne (2008)	28/F	Clomipramine SR 4.5gr	0	AC/ GL	4 h	Bezoar/radiopaque image	favorable
Höjer and Personne (2008)	25/F	Clomipramine SR 6gr Oxazepam 120 mgl	3	​	5.5 h	Bezoar in the esophagus	favorable
Johnson et al. (2017)	13/F	diphenhydramine 5gr	3	​	38 h	Bezoar/ radiopaque image	favorable
Inoue et al. (2023)	53/M	Paroxetine SR Quetiapine Trazodone, Aripiprazole Flunitrazepam Zopiclone	3	​	>10 h	bezoar with partial removal complicated by esophageal tear	death for MOF and ARDS
Parker Cote et al. (2022)	F/64y	Salicylate	3	AC	>120 h	packet/paper wrapped and bezoar/negative RX and TC	death for AKI and MOF
Djogovic et al. (2007)	47/F	Venlafaxine SR 15 gr	3	​	>120 h	bezoar	persistent come
Cereda et al. (1986)	18/F	Theophylline SR18 gr	3	GL AC	12H	bezoar	favorable
Tecklenburg et al. (2012)	16/ F	Bupropion Aripiprazole	3	AC	18 h	bezoar	favorable
von Düring et al. (2019)	40/F	Clomipramine Lorazepam Domperidone	3	AC	Unknown	Bezoar/radiopaque image	favorable
Wells et al. (2006)	61/F	Venlafaxine SR Nifedipine Atorvastatin Sertraline	3	AC GL	Unknown	Bezoar/ oral contrast under fluoroscopy and a cystic-appearing mass	death for MOF
Madan et al. (2021)	55/F	KCl 93 gr	3	​	6	Tablets/radiopaque image	favorable
Madan et al. (2021)	55/F	KCl 120gr	3	GL	12	Tablets/radiopaque image	favorable
Madan et al. (2021)	23 F	KCl 120 gr	3	WBI	4	Tablets/radiopaque image	favorable
Madan et al. (2021)	23F	KCl 36gr	3	​	Unknown	Tablets/radiopaque image	favorable
Miyauchi et al. (2013)	35/F	Amitriptyline 1.45gr Etizolam 18 m Triazolam 2.5 mg	3	AC GL	2	Tablets	favorable

AKI: acute kidney injury; MOF: multi organ failure.

Favorable outcomes were reported in 28 out of 32 published cases. Fatalities and prolonged comas were attributed to the toxicity of the ingested substances, not to the procedure itself. Complications were rare, with only one reported case of esophageal tears, which resolved without major sequelae. Bezoars were identified in 23 of the 32 cases (Table 3).

Based on both our data and previously published reports, decontaminative gastroscopy may have a critical role in selected toxic ingestion scenarios—particularly when involving SR formulations, bezoar-forming substances, or suspected lethal doses (Gosselin et al., 2025; Buckley et al., 1995; Adams et al., 2004; Iwamuro et al., 2015). It remains difficult to define a clear timeframe for the utility of EGD. Even in cases involving the same drug (e.g., Case 6 vs. Case 16) and similar ingested quantities, endoscopic findings varied significantly. An *in vitro* study by Lotte et al. showed that SR formulations can form bezoars that persist for up to 48 h before fully dissolving (Hoegberg et al., 2019). Our observations support the possibility that solid residues or bezoars may remain in the gastric lumen even more than 24 h post-ingestion.

While imaging modalities such as X-ray or CT scans can assist in identifying retained gastric contents, their utility is limited for non-radiopaque drugs (Guillermo et al., 2014; Sato and Kamijo, 2014; Gavala et al., 2017; Labarinas et al., 2018; McCarthy and Coleman, 2023; Briggs and Deal, 2014; Höjer and Personne, 2008; Johnson et al., 2017; von Düring et al., 2019; Wells et al., 2006; Madan et al., 2021). CT scans are further constrained by radiation exposure concerns and limited availability. Our center is currently exploring the potential role of abdominal ultrasound in detecting residual gastric materials.

Table 4 outlines drugs prone to bezoar formation and those that are radiopaque, based on our experience and prior literature. In many cases, multiple GD techniques were employed concurrently.

**TABLE 4:** Radiopaque drugs and drugs prone to form bezoar.

Radiopaque	Bezoar
Extended-release potassium chloride	Quetiapine SR
Vegetamine A	Vegetamine A
Methadone	Methadone
clomipramine SR	Diphenhydramine
Diphenhydramine	Clomipramine SR
	Salicylate
	Venlafaxine SR
	Theophylline SR
	Isoniazid
	Aripiprazole SR

We remain cautious about the use of GL. Published studies question its efficacy in removing high-risk ingested doses. Moreover, in cases involving bezoars, GL may break them apart, potentially increasing absorption and limiting the subsequent effectiveness of EGD. In our 15 cases where GL was performed prior to EGD, 12 patients still had significant amounts of bezoars, tablets, or drug-containing material.

At present, data do not support the routine use of gastroscopy in all poisoning cases. However, our findings suggest that EGD can be valuable in selected scenarios, particularly when performed by trained personnel and in the absence of contraindications (Gosselin et al., 2025; Nelson, 2000; Livshits et al., 2015; Buckley et al., 1995; Adams et al., 2004; Iwamuro et al., 2015; Marano et al., 2023). Though EGD may appear invasive, it could prevent the need for more aggressive interventions such as CVVHDF or ECMO in life-threatening overdoses. The availability of skilled practitioners is a key determinant for its use.

Pediatric patients pose unique challenges for GD, including weight-based dosing thresholds, the need for appropriately sized equipment, and the often uncooperative nature of younger children. While adult GD guidelines remain incomplete, evidence in pediatric populations is even more limited. Apart from our study, only seven published case reports describe EGD for decontamination in patients under 18 years (Labarinas et al., 2018; Allen et al., 2022; Johnson et al., 2017; Djogovic et al., 2007; Cereda et al., 1986; Marano et al., 2023; Marano et al., 2024).

This study presents several limitations that should be acknowledged. First, the retrospective study design and relatively small sample size limit the generalizability of the findings. Second, the absence of standardized guidelines to define the appropriateness of the procedure introduces a degree of subjectivity in the clinical decision-making process. This variability may have influenced both the selection of patients and the interpretation of endoscopic findings. Further research with larger cohorts and clearer procedural indications is needed to validate these preliminary observations and to establish evidence-based protocols.

In summary, based on our experience with 19 pediatric patients and 32 previously reported cases, EGD may be a useful tool in managing specific poisonings—particularly those involving massive ingestion, SR formulations, GI motility-impairing substances, or in cases where life-threatening ingestion cannot be ruled out. Our findings underscore the need to better define the role of gastroscopy in clinical toxicology, including standardized indications and contraindications. Further studies are essential to develop evidence-based guidelines for its integration into poisoning management protocols.

## Data Availability

The original contributions presented in the study are included in the article/supplementary material, further inquiries can be directed to the corresponding author.
